# An image interaction approach to quantum-phase engineering of two-dimensional materials

**DOI:** 10.1038/s41467-022-32508-5

**Published:** 2022-09-02

**Authors:** Valerio Di Giulio, P. A. D. Gonçalves, F. Javier García de Abajo

**Affiliations:** 1 ICFO-Institut de Ciencies Fotoniques, The Barcelona Institute of Science and Technology, 08860 Castelldefels, Barcelona Spain; 2 ICREA-Institució Catalana de Recerca i Estudis Avançats, Passeig Lluís Companys 23, 08010 Barcelona, Spain

**Keywords:** Two-dimensional materials, Electronic properties and materials

## Abstract

Tuning electrical, optical, and thermal material properties is central for engineering and understanding solid-state systems. In this scenario, atomically thin materials are appealing because of their sensitivity to electric and magnetic gating, as well as to interlayer hybridization. Here, we introduce a radically different approach to material engineering relying on the image interaction experienced by electrons in a two-dimensional material when placed in proximity of an electrically neutral structure. We theoretically show that electrons in a semiconductor atomic layer acquire a quantum phase resulting from the image potential induced by the presence of a neighboring periodic array of conducting ribbons, which in turn modifies the optical, electrical, and thermal properties of the monolayer, giving rise to additional interband optical absorption, plasmon hybridization, and metal-insulator transitions. Beyond its fundamental interest, material engineering based on the image interaction represents a disruptive approach to tailor the properties of atomic layers for application in nanodevices.

## Abstract (web summary)

Existing approaches to modulating the properties of 2D materials typically involve heterostructuring or exposure to external fields. Here, the authors propose a gate-free non-contact approach to tuning the properties of a 2D semiconductor via the image interaction due to proximity to a neutral patterned structure.

## Introduction

At the heart of condensed-matter physics is the drive to manipulate materials in a purposeful fashion to improve or enable functionalities. To that end, the sustained advances in fabrication capabilities along with the continuous emergence of novel material platforms have together fueled the field over the past half-century. Moreover, when suitably engineered, nanostructured materials can generally exhibit new properties beyond those found in their native, bulk form. A paradigmatic example that benefited from such approach was the development of semiconductor devices^1,2^, whose electronic properties were controlled by modifying the band structure through, for example, spatially patterning their compositional or doping characteristics^2–7^.

With the advent of two-dimensional (2D) and atomically thin materials^8–11^, those ideas were swiftly transferred to this arena as well, leading to the realization of 2D superlattices that incorporated not only vertical stacks, but also laterally assembled heterostructures^12–17^, as well as electrically modulated graphene^18,19^, artificial graphene^20,21^, and optical near-field dressing through periodic patterning of the supporting dielectric substrate^22^. More recently, similar band structure engineering concepts have been explored to create moiré superlattices^12,23–26^ and moiré excitons^27–29^, and also to investigate topological phenomena^30–33^. However, these approaches are generally invasive, as they require physical material nanostructuring, the injection of charge carriers, or interlayer hopping. Now, the question arises, can a more gentle engineering of a 2D material be realized without structural modifications or exposure to external fields?

**Fig. 1:**
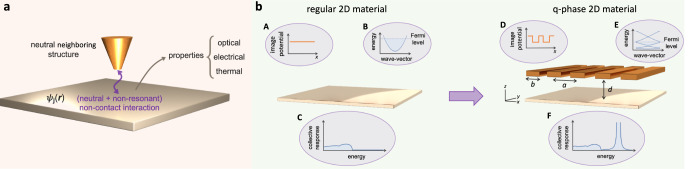
Realization of a quantum-phase (Q-phase) material. **a** Sketch of the non-contact interaction between a two-dimensional (2D) material and a neighboring neutral structure. The image potential experienced by conduction electrons in the material imprints a Q-phase on their wave functions *ψ*_*j*_(**r**) that in turn changes the optical, electrical, and thermal transport properties. **b** Possible realization of a Q-phase material. In the absence of an additional structure, there is no image interaction (A), so conduction electrons exhibit a characteristic parabolic dispersion (B), leading to a collective response function like that of a 2DEG (C). An image potential landscape (D) is produced by introducing a neighboring neutral structure (a periodic array of conducting ribbons of period *a*, width *b*, and separation *d*). The electron wave functions then acquire a Q-phase that reshapes the band structure, opening gaps (E) and enabling additional electronic transitions that translate into modifications of the material properties (F), including its collective excitations.

In this work, we introduce a disruptive approach for tailoring the electrical, optical, and thermal properties of 2D materials based on the manipulation of their electronic band structures by means of the gate-free, non-contact image-potential interaction experienced by the electrons of the material in the presence of a neutral neighboring structure (see Fig. 1). Indeed, when a charged particle (e.g., an electron in the 2D material) is placed near an interface, an image potential is induced that affects the particle dynamics. For free electrons, the image interaction is tantamount to the position-dependent Aharonov–Bohm quantum phase (Q-phase) imprinted on their wave functions by the self-induced electromagnetic fields in the vicinity of the interface^34^. Likewise, valence and conduction electrons in a material should acquire a Q-phase that depends on the geometrical and compositional details of the environment. This phase is expected to modify the electronic energy bands and, consequently, the dynamical response and transport properties of the hosting material. In reference to the origin of these modifications, hereinafter we refer to such 2D-material-based configurations as Q-phase materials.

Here, we introduce a specific realization of Q-phase materials consisting of a 2D semiconductor that is modified by the presence of a one-dimensional (1D) periodic array of ribbons. Specifically, we demonstrate that the periodic image potential produced by this pattern on the semiconductor electrons gives rise to substantial modifications in its band structure that translate into changes in the optical, electrical, and thermal properties, which are controlled by purely geometrical parameters (the separation and period of the array, see Fig. 1). We present rigorous theoretical calculations of the self-consistent electronic band structure, the ensuing optical response, and the electrical and thermal conductivities, all of which reveal dramatic modulations due to the aforementioned image interaction. More precisely, we predict the opening of electronic gaps, which enable interband optical transitions and hybrid plasmon-interband modes that would otherwise be optically forbidden in the absence of the image interaction. In addition, we observe a metal-insulator transition as the pattern is brought closer to the semiconductor and the image interaction increases, essentially reflecting a reduction in carrier propagation induced by the periodic patterning. This also affects the thermal conductivity, which we predict to undergo a corresponding conductor-insulator transition (along the direction of the periodic modulation, perpendicular to the ribbons). Importantly, the image interaction is long-range and nonresonant, so this method can be generally applied to any 2D material, adding a brand new tool to design nanodevices.

## Results

### Theoretical framework

We consider an atomically thin semiconductor of area *L*^2^, doped to a Fermi level ${E}_{{{{{{{{\rm{F}}}}}}}}}^{0}$ (corresponding to a carrier density *n*_0_ in the conduction band), and lying in the *z* = 0 plane at a distance *d* > 0 from a 1D periodic array of conductive ribbons (period *a*, width *b*, see Fig. 1b). The latter are taken to be infinitely extended along the *y* direction, periodic along *x*, and made of a material with a high DC conductivity and low infrared absorbance (e.g., indium tin oxide^35^), so that it behaves as a perfect conductor with regards to the determination of the equilibrium configuration of the electronic structure in the semiconductor. A patterned and doped semiconductor monolayer with a partially filled conduction band could serve this purpose. We further consider the periodic array to be embedded in a medium of permittivity *ϵ* matching that of the ribbon material at the optical frequencies discussed below, such that the array appears to be invisible. The semiconductor electrons can however interact with the array through a image potential energy −*V*_0_^36–38^, which for distances *d* ≳ 1 nm can be well approximated by the local electrostatic limit^39–41^ (i.e., *V*_0_ = *e*^2^/4*ϵd*). Incidentally, films consisting of three atomic layers of hexagonal boron nitride are now commonly used to introduce a dielectric spacing of ∼1 nm in 2D materfial heterostructures^42^, but other materials amenable to exfoliation down to a few monolayers could also be employed for that purpose, as well as atomic layer deposition^43^ methods. Neglecting ribbon edge effects by assuming *d* ≪ *a*, we describe the image interaction experienced by each semiconductor electron through the periodic stepwise energy function ${V}^{{{{{{{{\rm{im}}}}}}}}}(x)=-\!{V}_{0}p(x)$, where *p*(*x*) = 1 for *x* directly below a ribbon and *p*(*x*) = 0 otherwise. In this work, we set *ϵ* = 1 for simplicity and remark that the image potential is the result of the Q-phase introduced in the electron wave functions due to their interaction with the surrounding patterned structure, as illustrated for free electrons moving near a material surface^34^.

We describe the semiconductor electrons in the single-particle approximation^44^, and further consider them to be strongly confined to an atomic layer of small thickness compared with the separation *d* from the conductive ribbons. This allows us to factorize the one-electron wave functions as ${\psi }_{{{{{{{{\bf{k}}}}}}}}_{\parallel} n}({{{{{{{\bf{r}}}}}}}})={\psi }_{{{{{{{{{\bf{k}}}}}}}}}_{\parallel }n}({{{{{{{\bf{R}}}}}}}}){\psi }_{\perp }(z)$, with out-of-plane components yielding a probability density profile ∣*ψ*_⊥_(*z*)∣^2^ ≈ *δ*(*z*). The remaining in-plane components depend on **R** = (*x*, *y*) and are determined by the self-consistent equation^44^

1 $$\left[{{{{{{{{\mathcal{H}}}}}}}}}^{0}+{V}^{{{{{{{{\rm{im}}}}}}}}}+{V}^{{{{{{{{\rm{H}}}}}}}}}\right]{\psi }_{{{{{{{{{\bf{k}}}}}}}}}_{\parallel }n}({{{{{{{\bf{R}}}}}}}})=\hslash {\varepsilon }_{{{{{{{{{\bf{k}}}}}}}}}_{\parallel }n}{\psi }_{{{{{{{{{\bf{k}}}}}}}}}_{\parallel }n}({{{{{{{\bf{R}}}}}}}}),$$

where ${{{{{{{{\mathcal{H}}}}}}}}}^{0}=-{\hslash }^{2}{{{{{{\nabla }}}}}}_{{{{{{{{\bf{R}}}}}}}}}^{2}/2{m}^{*}$ is the unperturbed electron Hamiltonian, m^*^ denotes the effective mass, ${V}^{{{{{{{{\rm{im}}}}}}}}}$ is the aforementioned image potential, and ${V}^{{{\rm{H}}}}$ is the Hartree potential. Also, in virtue of Bloch’s theorem, the electron eigenstates can be labeled by the in-plane wave vector **k**_∥_ and the band index *n*, such that ${\psi }_{{{{{{{{{\bf{k}}}}}}}}}_{\parallel }n}({{{{{{{\bf{R}}}}}}}})={{{{{{{{\rm{e}}}}}}}}}^{{{{{{{{\rm{i}}}}}}}}{{{{{{{{\bf{k}}}}}}}}}_{\parallel }\cdot {{{{{{{\bf{R}}}}}}}}}\,{u}_{{{{{{{{{\bf{k}}}}}}}}}_{\parallel }n}({{{{{{{\bf{R}}}}}}}})/L$, where ${u}_{{{{{{{{{\bf{k}}}}}}}}}_{\parallel }n}({{{{{{{\bf{R}}}}}}}})={u}_{{{{{{{{{\bf{k}}}}}}}}}_{\parallel }n}({{{{{{{\bf{R}}}}}}}}+\ell a\hat{{{{{{{{\bf{x}}}}}}}}})$ for any integer ${\ell}$. In particular, given the translational invariance of the system under consideration along the *y* direction and the periodicity of the image potential along *x*, we have (see Supplementary Note 1)

2a $${\psi }_{{{{{{{{{\bf{k}}}}}}}}}_{\parallel }n}({{{{{{{\bf{R}}}}}}}})={{{{{{{{\rm{e}}}}}}}}}^{{{{{{{{\rm{i}}}}}}}}{{{{{{{{\bf{k}}}}}}}}}_{\parallel }\cdot {{{{{{{\bf{R}}}}}}}}}{u}_{{k}_{x}n}(x)/L,$$

2b $${\varepsilon }_{{{{{{{{{\bf{k}}}}}}}}}_{\parallel }n}={\varepsilon }_{{k}_{x}n}^{x}+\hslash {k}_{y}^{2}/2{m}^{*},$$

where ${u}_{{k}_{x}n}$ and ${\varepsilon }_{{k}_{x}n}^{x}$ are directly obtained by projecting Eq. (1) on Fourier components (i.e., by expanding the potentials and the wave function in the form *f*(*x*) = ∑ _*G*_ *f* _*G*_ e^i*Gx*^, where the sum extends over reciprocal lattice vectors *G* that are multiples of 2*π*/*a*, as detailed in Methods and in the Supplementary Note 1).

We solve Eq. (1) iteratively by partially updating the Hartree potential ${V}^{{{{{{{{\rm{H}}}}}}}}}({{{{{{{\bf{R}}}}}}}})={e}^{2}\int {d}^{2}{{{{{{{{\bf{R}}}}}}}}}^{\prime}\,\left[n({{{{{{{{\bf{R}}}}}}}}}^{\prime})-{n}_{0}\right]/|{{{{{{{\bf{R}}}}}}}}-{{{{{{{{\bf{R}}}}}}}}}^{\prime}|$ at each step, introducing the electron density $n({{{{{{{\bf{R}}}}}}}})=2{\sum }_{{{{{{{{{\bf{k}}}}}}}}}_{\parallel }n}\,{f}_{{{{{{{{{\bf{k}}}}}}}}}_{\parallel }n}|{\psi }_{{{{{{{{{\bf{k}}}}}}}}}_{\parallel }n}({{{{{{{\bf{R}}}}}}}}){|}^{2}$ calculated from the (spin-degenerate) electron wave functions and the Fermi–Dirac distribution ${f}_{{{{{{{{{\bf{k}}}}}}}}}_{\parallel }n}$ [e.g., ${f}_{{{{{{{{{\bf{k}}}}}}}}}_{\parallel }n}=\theta ({E}_{{{{{{{{\rm{F}}}}}}}}}-\hslash {\varepsilon }_{{{{{{{{{\bf{k}}}}}}}}}_{\parallel }n})$ at *T* = 0K]. Importantly, we assume the semiconductor to remain electrically neutral in its environment, so that no charge imbalance is introduced by external dopants. Then, the charge neutrality condition $\int {d}^{2}{{{{{{{\bf{R}}}}}}}}\,\left[n({{{{{{{\bf{R}}}}}}}})-{n}_{0}\right]=0$ needs to hold at every iteration step and is used to determine the Fermi energy *E*_F_, which generally deviates from the value ${E}_{{{{{{{{\rm{F}}}}}}}}}^{0}$ in the homogeneous semiconductor (i.e., in the absence of image interaction). Incidentally, we note that the parabolic band assumed to describe the unperturbed conduction band of the doped semiconductor should be an excellent approximation because the patterning period *a* exceeds by several orders of magnitude the interatomic distance, thus leading to a comparatively small 1D first Brillouin zone (1BZ) of extension 2π/*a*.

### Modulation of the electronic band structure

**Fig. 2:**
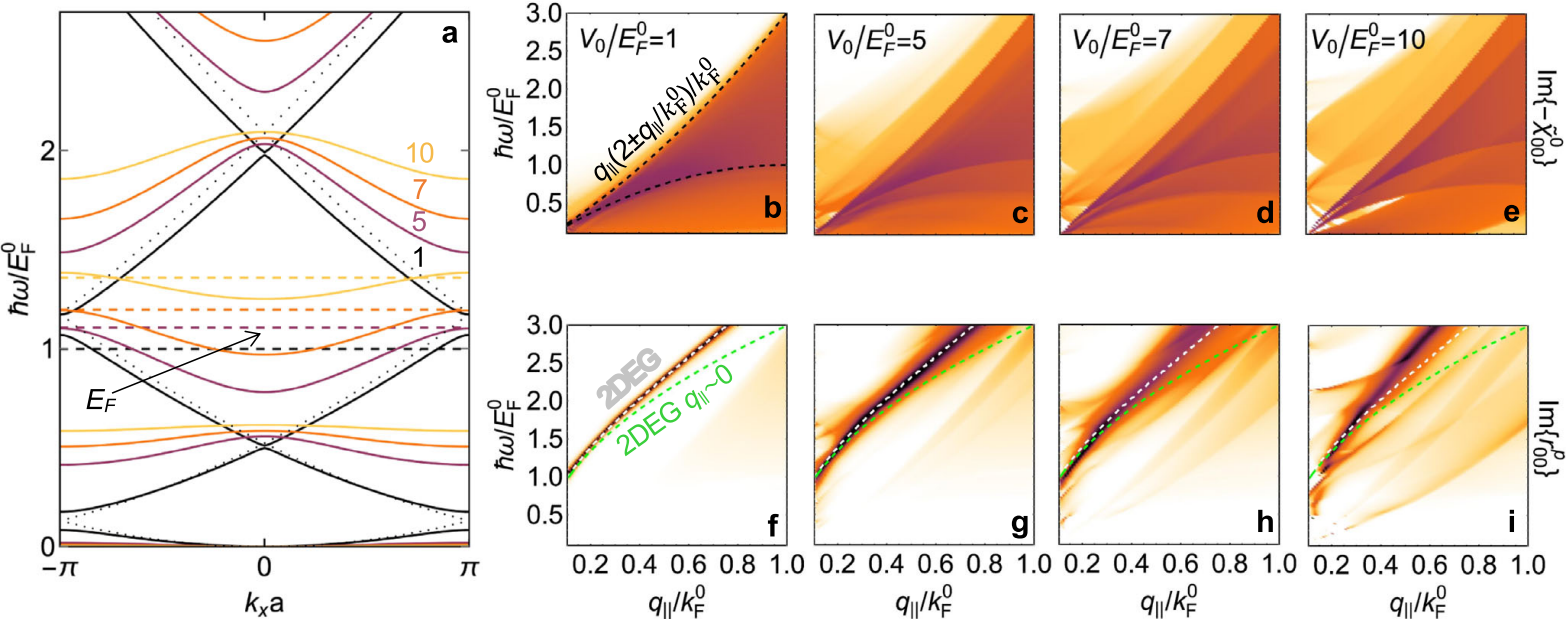
Modulation of the electronic band structure and optical response in a Q-phase material. **a** Electronic bands (solid curves) in the periodic patterning direction for different strengths of the normalized image potential ${V}_{0}/{E}_{{{{{{{{\rm{F}}}}}}}}}^{0}$ (see color-matched labels), along with the corresponding normalized Fermi energies (dashed lines). Dotted curves stand for the 2D-electron-gas (2DEG) limit. Single-particle excitations described by the 2D noninteracting susceptibility ${\tilde{\chi }}_{00}^{0}$ (**b**–**e**) and collective response resonances revealed by the loss function ${{{{{{{\rm{Im}}}}}}}}\{{r}_{00}^{{{{{{{{\rm{p}}}}}}}}}\}$ (**f**–**i**) as a function of transferred energy *ℏω* and in-plane wave vector *q*_∥_ = ∣(*q*_*x*_, *q*_*y*_)∣, normalized to the Fermi energy ${E}_{{{{{{{{\rm{F}}}}}}}}}^{0}$ and wave vector ${k}_{{{{{{{{\rm{F}}}}}}}}}^{0}$, respectively, for fixed ${q}_{x}/{k}_{{{{{{{{\rm{F}}}}}}}}}^{0}=0.1$. In **b**, the black-dashed curves indicate the boundaries of the continuum of electron-hole pair excitations $\hslash \omega /{E}_{{{{{{{{\rm{F}}}}}}}}}^{0}={q}_{\parallel }(2\pm {q}_{\parallel }/{k}_{{{{{{{{\rm{F}}}}}}}}}^{0})/{k}_{{{{{{{{\rm{F}}}}}}}}}^{0}$. The plasmon dispersion relation in a 2DEG is shown for comparison in **f**–**i** (white-dashed curves), along with its ${q}_{\parallel }/{k}_{{{{{{{{\rm{F}}}}}}}}}^{0}\ll 1$ limit (green-dashed curves). All calculations are performed for ${E}_{{{{{{{{\rm{F}}}}}}}}}^{0}=0.29$ eV, m^*^/*m*_e_ = 0.1, *a* = 10 nm, and *b* = 5 nm.

Once the conducting ribbons are brought close to the semiconductor, electrons in the latter are no longer free to move along the patterning direction *x* because they are modulated by the self-induced potential resulting from the image interaction. In particular, the electronic band structure is modified by the emergence of band gaps at the center and edges of the 1BZ (see Fig. 2a), which also imply changes in the electron velocity component ${\partial }_{{k}_{x}}{\varepsilon }_{{k}_{x}n}^{x}\equiv {v}_{{k}_{x}n}$ and the effective mass. We note that the normalized band energies $\hslash {\varepsilon }_{{k}_{x}n}^{x}/{E}_{{{{{{{{\rm{F}}}}}}}}}^{0}$ and the dimensionless eigenvectors $a{\psi }_{{{{{{{{{\bf{k}}}}}}}}}_{\parallel }n}({{{{{{{\bf{R}}}}}}}})$ depend only on four independent parameters: (1) the geometrical ratio *b*/*a* (see Fig. 1b), which regulates the *tunneling rate* across barriers introduced by the image potential; (2) the size of the 1BZ *k*_BZ_ = *π*/*a* relative to the unperturbed Fermi wave vector (i.e., ${k}_{{{{{{{{\rm{BZ}}}}}}}}}/{k}_{{{{{{{{\rm{F}}}}}}}}}^{0}$); (3) the strength of the image energy relative to the unperturbed Fermi energy, ${V}_{0}/{E}_{{{{{{{{\rm{F}}}}}}}}}^{0}$; and (4) the normalized electron-electron Coulomb interaction energy across a unit cell ${V}_{{{{{{{{\rm{C}}}}}}}}}/{E}_{{{{{{{{\rm{F}}}}}}}}}^{0}$, where *V*_C_ = *e* ^2^/*a*. As illustrated in the dispersion diagrams shown in Fig. 2a for different values of ${V}_{0}/{E}_{{{{{{{{\rm{F}}}}}}}}}^{0}$, when the ribbons are moved far apart, and thus ${V}_{0}/{E}_{{{{{{{{\rm{F}}}}}}}}}^{0}$ approaches 0, we recover a folded 2DEG (2D electron gas, dotted curves) at the center of the 1BZ, while small gaps of decreasing size remain visible at the zone edge. Reassuringly, we find that for a vanishing value of ${V}_{{{{{{{{\rm{C}}}}}}}}}/{E}_{{{{{{{{\rm{F}}}}}}}}}^{0}$ the Hartree potential contributes negligibly to the energy of the system and the eigenvalues ${\varepsilon }_{{k}_{x}n}^{x}$ agree well with those obtained in the Krönig–Penney model^45^. In addition, when varying the geometrical ratio *b*/*a*, we find oscillations in the magnitude of the band gap produced by the image-interaction modulation (see Methods), with the extreme cases of *b*/*a* = 0 and 1 reducing to just a rigid shift in the energy bands (see Supplementary Fig. 2 and Supplementary Note 1).

Here, we are interested in a regime where the image potential strongly affects the transport properties of the material. Such a regime is reached when the total (i.e., summed for all electrons) image energy ${E}^{{{{{{{{\rm{im}}}}}}}}}$ dominates over the kinetic energy *E*^kin^, as otherwise the latter would push the system toward a ballistic behavior. The ratio between these two energies roughly scales as ${E}^{{{{{{{{\rm{im}}}}}}}}}/{E}^{{{{{{{{\rm{kin}}}}}}}}} \sim {V}_{0}/{E}_{{{{{{{{\rm{F}}}}}}}}}^{0}$, assuming that the condition $({V}_{{{{{{{{\rm{C}}}}}}}}}/{E}_{{{{{{{{\rm{F}}}}}}}}}^{0})/{({k}_{{{{{{{{\rm{BZ}}}}}}}}}/{k}_{{{{{{{{\rm{F}}}}}}}}}^{0})}^{2}=(2/{\pi }^{2})\,{e}^{2}{m}^{*}a/{\hslash }^{2}\,\gg \,1$ is satisfied (e.g., for *a* ≫ 2.6 nm if m^*^ = 0.1 *m*_e_, see Supplementary Note 2). This behavior is observed in the band structure calculations presented in Fig. 2a, where the influence of the image potential is visible through a monotonic increase in the band gaps with increasing strength of the image interaction *V*_0_ (i.e., when bringing the ribbon pattern closer to the semiconductor). The reshaping of the semiconductor energy bands also produces a wealth of new dynamical and static properties, which we investigate below. It should be noted that, in order to open a band gap ${V}_{{{{{{{{\rm{gap}}}}}}}}} \sim {k}_{{{{{{{{\rm{BZ}}}}}}}}}{V}_{0}/{k}_{{{{{{{{\rm{F}}}}}}}}}^{0}$ in the infrared range (e.g., 0.1–0.2 eV) using a ratio ${V}_{0}/{E}_{{{{{{{{\rm{F}}}}}}}}}^{0} \sim 3$ and an image potential energy *V*_0_ ∼ 0.3 eV (requiring a patterning distance *d* ∼ 1.2 nm) in combination with a period *a* = 10 nm, materials with m^*^/*m*_e_ ∼ 0.1–0.4 are needed (see, for example, Fig. 2a and Supplementary Fig. 4a). Such values are found in black phosphorus^46^ and transition-metal dichalcogenides^47^, from which monolayers can be isolated for a direct implementation of the concepts explored here.

### Optical response of Q-phase materials

The modifications produced in the electronic band structure by the image interaction translate into substantial changes in the optical response. Given the symmetry of the system, we can work in the in-plane reciprocal space and separately deal with each 2D wave vector **q**_∥_ (with *q*_*x*_ within the 1BZ). In addition, our structures involve small distances and periods compared with the light wavelength, so that the optical response can be well described in the electrostatic limit. Consequently, we consider an external electric potential ${\phi }^{{{{{{{{\rm{ext}}}}}}}}}({{{{{{{{\bf{q}}}}}}}}}_{\parallel },z,\omega )={\sum }_{G}{\phi }_{G}^{{{{{{{{\rm{ext}}}}}}}}}({{{{{{{{\bf{q}}}}}}}}}_{\parallel },z,\omega ){{{{{{{{\rm{e}}}}}}}}}^{{{{{{{{\rm{i}}}}}}}}({{{{{{{{\bf{q}}}}}}}}}_{\parallel }+G\hat{{{{{{{{\bf{x}}}}}}}}})\cdot {{{{{{{\bf{R}}}}}}}}}$ acting on the Q-phase material at optical frequency *ω* and expanded in Fourier components labeled by 1D reciprocal lattice vectors *G*. Introducing a matrix notation, we can express the resulting induced potential in terms of the Coulomb interaction *υ* and the noninteracting 2D susceptibility ${\tilde{\chi }}^{0}$ as ${\phi }^{{{{{{{{\rm{ind}}}}}}}}}({{{{{{{{\bf{q}}}}}}}}}_{\parallel },\,z,\,\omega )={\upsilon }({{{{{{{{\bf{q}}}}}}}}}_{\parallel },\,z)\cdot {\tilde{\chi }}^{0}({{{{{{{{\bf{q}}}}}}}}}_{\parallel },\,\omega )\cdot {[{{{{{{{\mathcal{I}}}}}}}}-\upsilon ({{{{{{{{\bf{q}}}}}}}}}_{\parallel },\,0)\cdot {\tilde{\chi }}^{0}({{{{{{{{\bf{q}}}}}}}}}_{\parallel },\,\omega )]}^{-1}\cdot {\phi }^{{{{{{{{\rm{ext}}}}}}}}}({{{{{{{{\bf{q}}}}}}}}}_{\parallel },\,0,\,\omega )$, where dots indicate matrix multiplication, *ϕ*^ext^ and *ϕ*^ind^ are vectors of components ${\phi }_{G}^{{{{{{{{\rm{ext}}}}}}}}}$ and ${\phi }_{G}^{{{{{{{{\rm{ind}}}}}}}}}$, respectively, and we use the matrices ${{{{{{{{\mathcal{I}}}}}}}}}_{G{G}^{\prime}}={\delta }_{G{G}^{\prime}}$, ${\upsilon }_{G{G}^{\prime}}={\delta }_{G{G}^{\prime}}{\upsilon }_{G}$, and ${\tilde{\chi }}_{G{G}^{\prime}}^{0}$. More precisely, the diagonal Coulomb matrix elements take the form ${\upsilon }_{G}({{{{{{{{\bf{q}}}}}}}}}_{\parallel },z)=2\pi \,{{{{{{{{\rm{e}}}}}}}}}^{-|{{{{{{{{\bf{q}}}}}}}}}_{\parallel }+G\hat{{{{{{{{\bf{x}}}}}}}}}|\vert z|}/|{{{{{{{{\bf{q}}}}}}}}}_{\parallel }+G\hat{{{{{{{{\bf{x}}}}}}}}}|$, while we adopt the random-phase approximation (RPA) to evaluate the susceptibility from the one-electron eigenstates as^39,44^ (see Methods)

3 $${\tilde{\chi }}_{G{G}^{\prime}}^{0}({{{{{{{{\bf{q}}}}}}}}}_{\parallel },\,\omega )=	 \frac{{e}^{2}}{2{\pi }^{2}\hbar }\,\mathop{\sum}\limits_{n{n}^{\prime}}\int\nolimits_{-\pi /a}^{\pi /a}d{k}_{x}{M}_{G}^{n{n}^{\prime}}({k}_{x},\,{q}_{x}){\left[{M}_{{G}^{\prime}}^{n{n}^{\prime}}({k}_{x},\,{q}_{x})\right]}^{*}\\ 	 \times \int\nolimits_{-\infty }^{\infty }d{k}_{y}\frac{{f}_{{{{{{{{{\bf{k}}}}}}}}}_{\parallel }-{{{{{{{{\bf{q}}}}}}}}}_{\parallel }{n}^{\prime}}-{f}_{{{{{{{{{\bf{k}}}}}}}}}_{\parallel }n}}{\omega -{\varepsilon }_{{{{{{{{{\bf{k}}}}}}}}}_{\parallel }n}+{\varepsilon }_{{{{{{{{{\bf{k}}}}}}}}}_{\parallel }-{{{{{{{{\bf{q}}}}}}}}}_{\parallel }{n}^{\prime}}+{{{{{{{\rm{i}}}}}}}}{0}^{+}},$$

where we have introduced the matrix elements ${M}_{G}^{n{n}^{\prime}}({k}_{x},\,{q}_{x})=(1/a)\int\nolimits_{0}^{a}dx\,{{{{{{{{\rm{e}}}}}}}}}^{-{{{{{{{\rm{i}}}}}}}}Gx}\,{u}_{{k}_{x}-{q}_{x}{n}^{\prime}}^{*}(x)\,{u}_{{k}_{x}n}(x)$.

The energy differences in the denominator of Eq. (3) correspond to one-electron excitations, which show up as intraband ($n={n}^{\prime}$) and interband ($n\ne {n}^{\prime}$) transitions in the plots of ${\tilde{\chi }}_{G{G}^{\prime}}^{0}({{{{{{{{\bf{q}}}}}}}}}_{\parallel },\omega )$ presented in Fig. 2b–e (as well as in Supplementary Fig. 3, in which we separately show intraband and interband contributions, and in Supplementary Fig. 4b–e, where we explore different material parameters) as a function of photon energy *ℏω* and parallel wave vector *q*_∥_ for the $G={G}^{\prime}=0$ component and different strengths of the image interaction *V*_0_. For small *V*_0_, the dispersion diagram is dominated by the intraband excitation region that characterizes the conduction band of a doped semiconductor (i.e., the homogeneous 2DEG limit). As we increase *V*_0_ (see Fig. 2b–e), the gap openings discussed above (Fig. 2a) enable interband excitations, and in particular, vertical transitions become available due to the proximity of the ribbon array.

The RPA accounts for the self-consistent interaction with the induced potential, thereby resulting in collective electron excitations. To explore these so-called plasmons, we calculate the Fresnel reflection coefficient for p-polarized waves *r* ^p^, which relates the induced and external potentials via^48^  ${\phi }_{G}^{{{{{{{{\rm{ind}}}}}}}}}({{{{{{{{\bf{q}}}}}}}}}_{\parallel },0,\omega )=-{\sum }_{{G}^{\prime}}{r}_{G{G}^{\prime}}^{{{{{{{{\rm{p}}}}}}}}}({{{{{{{{\bf{q}}}}}}}}}_{\parallel },\omega )\,{\phi }_{{G}^{\prime}}^{{{{{{{{\rm{ext}}}}}}}}}({{{{{{{{\bf{q}}}}}}}}}_{\parallel },0,\omega )$. Using the formalism outlined above, the Fourier components of *r* ^p^ are given by

$${r}_{G{G}^{\prime}}^{{{{{{{{\rm{p}}}}}}}}}({{{{{{{{\bf{q}}}}}}}}}_{\parallel },\,\omega )={\frac{{{\mathcal{I}}}}{{{{\mathcal{I}}}}-{\left[\upsilon ({{{{{{{{\bf{q}}}}}}}}}_{\parallel },\,0) \,{\cdot}\, {\tilde{\chi }}^{0}({{{{{{{{\bf{q}}}}}}}}}_{\parallel },\, \omega )\right]}^{-1}} }\Bigg|_{G{G}^{\prime}}.$$

In Fig. 2f–i, we plot the loss function ${{{{{{{\rm{Im}}}}}}}}\{{r}_{G{G}^{\prime}}^{{{{{{{{\rm{p}}}}}}}}}\}$ obtained from this equation as a function of *q*_∥_ and *ω*. We note that even though we concentrate on the specular-reflection coefficient corresponding to $G={G}^{\prime}=0$, the condition ${k}_{{{{{{{{\rm{BZ}}}}}}}}}\,\ll \,{k}_{{{{{{{{\rm{F}}}}}}}}}^{0}$ requires the evaluation of *G* components up to $G\,\gg \,6{k}_{{{{{{{{\rm{F}}}}}}}}}^{0}$ in the *υ* and ${\tilde{\chi }}^{0}$ matrices to correctly account for transitions happening close to the Fermi surface.

Collective plasmon excitations are identified as intense features in Fig. 2f–i, which should be measurable through electron energy-loss spectroscopy, as we discuss in Supplementary Note 3 and Supplementary Fig. 1. We expect those features to be qualitatively similar over the range of material parameters explored in Supplementary Fig. 4f–i. For relatively small ${V}_{0}/{E}_{{{{{{{{\rm{F}}}}}}}}}^{0}$ (Fig. 2f), the dispersion diagram is dominated by a single, continuous plasmon band, in excellent agreement with the plasmon dispersion of the textbook uniform 2DEG (superimposed). This agreement reflects the fact that the plasmon behavior is mainly controlled by the average electron density *n*_0_, provided the external perturbation produced in the band structure by the periodic image potential is still weak (see the relatively small gap openings in Fig. 2a for ${V}_{0}={E}_{{{{{{{{\rm{F}}}}}}}}}^{0}$). Incidentally, at low *V*_0_ the plasmon is qualitatively well described by the dispersion relation ${\omega }_{{{{{{{{\rm{p}}}}}}}}}({q}_{\parallel }) \sim e\sqrt{n{q}_{\parallel }/{m}^{*}}$ obtained in the ${q}_{\parallel }/{k}_{{{{{{{{\rm{F}}}}}}}}}^{0}\,\ll \,1$ limit. In contrast, as the ribbon structure is brought closer to the semiconductor, so that the image potential energy increases and eventually dominates,  a zoo of excitations emerge in the dispersion diagram: besides the 2DEG plasmon, features associated with interband transitions and their hybridization with plasmons are revealed. In addition, all of these features are dressed by electron-electron interactions, leading to a blue shift of the plasmon relative to the 2DEG limit, as well as spectral shifts of the interband transitions relative to the undressed excitations depicted in Fig. 2b–e.

### Metal-insulator transition

Beyond the optical response, we expect the image interaction to also modify the static properties of Q-phase materials, including the DC electrical conductivity *σ*^DC^. We compute this quantity in the relaxation-time approximation^49^, introducing a phenomenological inelastic scattering time *τ* (see Methods), so the conductivity is uniquely determined by the band energies $\hslash {\varepsilon }_{{{{{{{{{\bf{k}}}}}}}}}_{\parallel }n}$ and the chemical potential, which we approximate by the Fermi energy *E*_F_ (Fig. 2a). We remark that this assumption safely holds for temperatures and doping levels such that ${k}_{{{{{{{{\rm{B}}}}}}}}}T\,\ll \,{E}_{{{{{{{{\rm{F}}}}}}}}}^{0}$, a condition that is satisfied over the range of parameters considered in this work. Because of the symmetry of the system, the 2 × 2 conductivity tensor should only contain diagonal *xx* and *yy* components (i.e., along directions parallel and perpendicular to the periodic modulation). In addition, the *yy* component remains unchanged with respect to the unperturbed semiconductor because the *x*-averaged electron density is conserved (i.e., ${\sigma }_{yy}^{{{{{{{{\rm{DC}}}}}}}}}=({e}^{2}\tau /{\pi }^{2}{m}^{*}){\sum }_{n}\int\nolimits_{-\pi /a}^{\pi /a}d{k}_{x}\int\nolimits_{0}^{\infty }d{k}_{y}{[{{{{{{{{\rm{e}}}}}}}}}^{(\hslash {\varepsilon }_{{{{{{{{{\bf{k}}}}}}}}}_{\parallel }n}-{E}_{{{{{{{{\rm{F}}}}}}}}})/{k}_{{{{{{{{\rm{B}}}}}}}}}T}+1]}^{-1}$, which reduces to ${\sigma }_{yy}^{{{{{{{{\rm{DC}}}}}}}}}={e}^{2}{E}_{{{{{{{{\rm{F}}}}}}}}}^{0}\tau /\pi {\hslash }^{2}$ at zero temperature). The conductivity along *x* can then be directly computed from the energy distribution (i.e., the band structure) in Eq. (2b) through the equation

4 $${\sigma }_{xx}^{{{{{{{{\rm{DC}}}}}}}}}=	 \frac{{e}^{2}\tau }{{\pi }^{2}\hbar }\,\mathop{\sum}\limits_{n}\int\nolimits_{-\pi /a}^{\pi /a}d{k}_{x}\int\nolimits_{0}^{\infty }d{k}_{y}\\ 	 \left({\partial }_{{k}_{x}}^{2}{\varepsilon }_{{k}_{x}n}^{x}\right){\left[{{{{{{{{\rm{e}}}}}}}}}^{(\hslash {\varepsilon }_{{{{{{{{{\bf{k}}}}}}}}}_{\parallel }n}-{E}_{{{{{{{{\rm{F}}}}}}}}})/{k}_{{{{{{{{\rm{B}}}}}}}}}T}+1\right]}^{-1}.$$

**Fig. 3:**
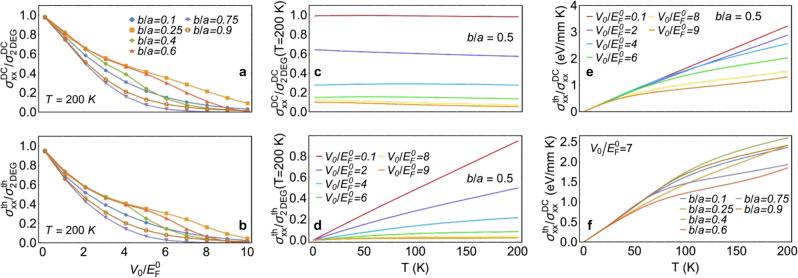
Q-phase modulation of the DC electrical and thermal conductivities. Component of the 2D electrical (**a**) and thermal (**b**) conductivity tensors along the periodicity direction as a function of the normalized image potential strength ${V}_{0}/{E}_{{{{{{{{\rm{F}}}}}}}}}^{0}$ for several values of the *b*/*a* ratio (see legend), calculated at *T* = 200 K and divided by the respective conductivities of a 2DEG at the same temperature. Temperature dependence of the electrical (**c**) and thermal (**d**) conductivities for *b*/*a* = 0.5 and different values of ${V}_{0}/{E}_{{{{{{{{\rm{F}}}}}}}}}^{0}$, normalized to the respective 2DEG conductivity at *T* = 200 K. Temperature dependence of the ratio between thermal and electric conductivities, revealing deviations from the Wiedemann–Franz law for *b*/*a* = 0.5 and various values of the ratio ${V}_{0}/{E}_{{{{{{{{\rm{F}}}}}}}}}^{0}$ (**e**), as well as for fixed ${V}_{0}/{E}_{{{{{{{{\rm{F}}}}}}}}}^{0}=5$ and several values of *b*/*a* (**f**). We use the same material parameters as in Fig. 2.

We use this expression in combination with the bands plotted in Fig. 2a to obtain the results presented in Fig. 3. Remarkably, the electrical conductivity exhibits a steady decrease with increasing image interaction *V*_0_, starting from the 2DEG value in the unperturbed semiconductor at *V*_0_ = 0 and evolving towards a substantial suppression when the *V*_0_/*E*_F_ ratio reaches a few times unity (see Fig. 3a). This behavior is a direct consequence of the reduction in the ability of charge carriers to move within the periodic potential landscape produced by the image interaction (due to the modulation of the electronic density brought about by the image potential). We thus predict a metal-insulator transition as the latter is switched on by placing the conductive ribbons closer to the semiconductor (i.e., the transition happens with respect to the order parameter ${V}_{0}/{E}_{{{{{{{{\rm{F}}}}}}}}}^{0}$, not to be confused with a phase transition driven by a change in temperature). This phenomenon is observed for all ribbon sizes under consideration, with an optimum behavior found for *b*/*a* ∼0.75, alongside a weak temperature dependence up to *T* = 200 K (see Fig. 3c).

### Inhibition of the thermal conductivity

Similar to the electric conductivity, the electronic thermal conductivity *σ* ^th^ undergoes strong modifications due to the image interaction, since it is equally mediated by carrier propagation. We adopt again the relaxation-time approximation^49^ and compute *σ*^th^ from the electronic band structure (see Methods). Following similar arguments as those outlined above, we find the conductivity tensor to be diagonal, with its *yy* component almost unaffected by the image interaction, so it can be well approximated by the relation ${\sigma }_{yy}^{{{{{{{{\rm{th}}}}}}}}}\approx \,(\pi {E}_{{{{{{{{\rm{F}}}}}}}}}^{0}{{k}_{{{{{{{{\rm{B}}}}}}}}}}^{2}T\tau /9{\hslash }^{2})[3-{(\pi {k}_{{{{{{{{\rm{B}}}}}}}}}T/{E}_{{{{{{{{\rm{F}}}}}}}}}^{0})}^{2}]$. The remaining *xx* component can be directly computed from

5 $${\sigma }_{xx}^{{{{{{{{\rm{th}}}}}}}}}=\frac{1}{{e}^{2}T}\left[{\sigma }_{xx}^{(2)}-{\left({\sigma }_{xx}^{(1)}\right)}^{2}/{\sigma }_{xx}^{{{{{{{{\rm{DC}}}}}}}}}\right],$$

where ${\sigma }_{xx}^{{{{{{{{\rm{DC}}}}}}}}}$ is the DC electrical conductivity in Eq. (4) and

6 $${\sigma }_{xx}^{{{{{{{{\rm{(\alpha )}}}}}}}}}=	 \frac{{e}^{2}\tau }{{\pi }^{2}{k}_{{{{{{{{\rm{B}}}}}}}}}T}\mathop{\sum}\limits_{n}\int\nolimits_{-\pi /a}^{\pi /a}d{k}_{x}\int\nolimits_{0}^{\infty }d{k}_{y}{\left(\hslash {\varepsilon }_{{{{{{{{{\bf{k}}}}}}}}}_{\parallel }n}-{E}_{{{{{{{{\rm{F}}}}}}}}}\right)}^{\alpha }\\ 	 \times {\left({\partial }_{{k}_{x}}{\varepsilon }_{{k}_{x}n}^{x}\right)}^{2}\frac{{{{{{{{{\rm{e}}}}}}}}}^{(\hslash {\varepsilon }_{{{{{{{{{\bf{k}}}}}}}}}_{\parallel }n}-{E}_{{{{{{{{\rm{F}}}}}}}}})/{k}_{{{{{{{{\rm{B}}}}}}}}}T}}{{\left[{{{{{{{{\rm{e}}}}}}}}}^{(\hslash {\varepsilon }_{{{{{{{{{\bf{k}}}}}}}}}_{\parallel }n}-{E}_{{{{{{{{\rm{F}}}}}}}}})/{k}_{{{{{{{{\rm{B}}}}}}}}}T}+1\right]}^{2}}.$$

The *V*_0_-dependent thermal conductivity plotted in Fig. 3b, d reveals a transition from a thermal conductor to a thermal insulator analogous to the electrical behavior both at low and at finite temperatures, which we also attribute to the reduction in carrier propagation produced by the periodic image potential. In addition, we observe a clear departure from the Wiedemann–Franz law^50^ when comparing Fig. 3a with Fig. 3b, as revealed by the different behavior at finite temperature displayed by ${\sigma }_{xx}^{{{{{{{{\rm{th}}}}}}}}}$ as a function of *V*_0_ with respect to ${\sigma }_{xx}^{{{{{{{{\rm{DC}}}}}}}}}$. Such deviation from the linear regime, which is also highlighted by the ratio between thermal and electrical conductivities plotted in Fig. 3e, f for several image potential strengths and different *b*/*a* ratios, stems from the fact that conduction electrons are not free, in contrast to the ideal-gas conditions for which the law is best suited. Incidentally, the thermal conductor-insulator transition is again faster for *b*/*a* ∼0.75.

## Discussion

In conclusion, we have demonstrated, based on rigorous theory, that the optical, electrical, and thermal properties of a 2D material can be substantially modified by introducing a neutral, non-contact structure in its vicinity. This constitutes a genuinely radical departure from currently available methods to engineer material properties, as instead we capitalize on the image interaction, which translates into a quantum phase imprinted on the conduction electrons of the 2D layer. We remark the nonresonant nature of such interaction, which should therefore be generally applicable to modulate the properties of different types of materials, provided their thickness is small enough as to be strongly influenced by the image interaction with the added structure.

As a possible realization of these engineered media, which we term Q-phase materials, we have studied the modification in the properties of a 2D semiconductor when an array of conductive ribbons is brought in close proximity. (Incidentally, dielectric ribbons with a high permittivity contrast should also produce a sufficiently intense image potential and serve as decorating elements for Q-phase materials.) Specifically, energy gaps are induced in the electronic band structure, interband electronic transitions are enabled, a rich landscape of additional plasmon bands emerges, and the electrical/thermal conductivity displays a metal/conductor-insulator transition as the semiconductor-array distance is reduced and the image interaction is increased. In the limits of either small ribbons (e.g., *b*/*a* = 0.1 in Fig. 3a, b, f) or a ribbon width approaching the period (e.g., *b*/*a* = 0.9), we obtain a spatially featureless image interaction, therefore resulting in a relatively small modulation of the material, and consequently, an optimized effect is observed at intermediate values of the width-to-period ratio *b*/*a* for a fixed separation from the 2D material.

In passing, we note that a transition of different nature is expected when the 2D semiconductor is in contact with the patterned structure (e.g., through structural reconstruction or via electron hopping), while here we are concerned instead with transitions occurring without touching or hopping, in which the order parameter is the strength of the image interaction. The predicted modifications in the optical, electrical, and thermal properties of the 2D material are driven by a rearrangement of its electronic bands in the presence of the image potential landscape, without requiring any physical contact. However, the strain caused by the structure could also play a role and introduce additional modifications in the material properties, although we expect this effect to be comparatively small when the 2D material and the patterned structure are separated by at least a few monolayers through which any possible microscopic reconstructions are attenuated.

As a practical realization of the present concept, materials such as black phosphorus^46^ and transition-metal dichalcogenides^47^ offer parameters similar to those considered above for the 2D layer. In addition, application of this scheme to graphene is anticipated to produce qualitatively different features due to the exotic nature of charge carriers in this material (e.g., electronic bands would be primarily renormalized in the direction parallel to the ribbons, in contrast to the results presented above, see Supplementary Note 4). Furthermore, the decorating structure could be amenable to modulation through external means of control (e.g., by resorting to phase-change materials such as GST^51^), still without having physical contact with the 2D layer. Although we focus here on large features of the decorating structure compared with the atomic lattice parameters of the material, so that conduction electrons in the latter are well described in the continuum limit, an interesting regime comes about when the characteristic patterning length is commensurate with the atomic lattice (e.g., by using a self-organized vicinal surface^52^ stamp). From a more general perspective, arbitrarily patterned external structures could be employed to, for example, shape the Q-phase material into quasicrystals, and even to draw electrical and thermal circuits controlled by the presence of a nontouching structure. Our results represent a first step towards the realization of gate-free material tunability, potentially granting us access into a whole range of properties that could find application in the design of nanodevices.

## Methods

### Self-consistent one-electron wave functions

We note that Eq. (1) can be solved by factorizing the one-electron wave functions as ${\psi }_{{{{{{{{{\bf{k}}}}}}}}}_{\parallel }n}({{{{{{{\bf{R}}}}}}}})={\psi }_{{k}_{x}n}(x){\varphi }_{{k}_{y}}(\,y)$, leading to

6a $$\left(\frac{{\hslash }^{2}}{2{m}^{*}}{\partial }_{y}^{2}+\hslash {\varepsilon }_{{k}_{y}n}^{y}\right)\,{\phi }_{{k}_{y}}(\,y)=0,$$

6b $$\left[\frac{{\hslash }^{2}}{2{m}^{*}}{({k}_{x}-{{{{{{{\rm{i}}}}}}}}{\partial }_{x})}^{2}+{V}_{{{{{{{{\rm{im}}}}}}}}}(x)+{V}_{{{{{{{{\rm{H}}}}}}}}}(x)-\hslash {\varepsilon }_{{k}_{x}n}^{x}\right]{u}_{{k}_{x}n}(x)=0,$$

where we have used Bloch’s theorem to express ${\psi }_{{k}_{x}n}(x)={{{{{{{{\rm{e}}}}}}}}}^{{{{{{{{\rm{i}}}}}}}}{k}_{x}x}{u}_{{k}_{x}n}(x)/\sqrt{L}$ in terms of a periodic function ${u}_{{k}_{x}n}(x)$ with the same period *a* as the ribbon array. From Eq. (6a), the component along the direction of translational invariance admits plane-wave solutions ${\phi }_{{k}_{y}}(\,y)={{{{{{{{\rm{e}}}}}}}}}^{{{{{{{{\rm{i}}}}}}}}{k}_{y}y}/\sqrt{L}$ with parabolic dispersion. In the remaining in-plane direction, Eq. (6b) transforms into an eigenvalue problem by expanding ${u}_{{k}_{x}n}(x)={\sum }_{G}{{{{{{{{\rm{e}}}}}}}}}^{{{{{{{{\rm{i}}}}}}}}Gx}{u}_{{k}_{x}n,G}$ as a sum over reciprocal lattice vectors *G* (multiples of 2*π*/*a*). More precisely, Eq. (6b) becomes

7 $$\left[\frac{{\hslash }^{2}}{2{m}^{*}}{\left({k}_{x}+G\right)}^{2}-\hslash {\varepsilon }_{{k}_{x}n}^{x}\right]{u}_{{k}_{x}n,G}+\mathop{\sum}\limits_{{G}^{\prime}}\left({V}_{G-{G}^{\prime}}^{{{{{{{{\rm{im}}}}}}}}}+{V}_{G-{G}^{\prime}}^{{{{{{{{\rm{H}}}}}}}}}\right){u}_{{k}_{x}n,{G}^{\prime}}=0,$$

where ${V}_{G}^{{{{{{{{\rm{im}}}}}}}}}={{{{{{{\rm{i}}}}}}}}\,\left({V}_{0}/aG\right)\left[1-{{{{{{{{\rm{e}}}}}}}}}^{-{{{{{{{\rm{i}}}}}}}}Gb}\right]$ and ${V}_{G}^{{{{{{{{\rm{H}}}}}}}}}=2\pi {e}^{2}(1-{\delta }_{G,0}){n}_{G}/|G|$ are the Fourier components of the image and Hartree potentials, respectively (see Supplementary Note 1). Finally, we solve Eq. (7) iteratively by calculating the components of the electron density ${n}_{G}=(2/a)\int\nolimits_{0}^{a}dx\,{{{{{{{{\rm{e}}}}}}}}}^{-{{{{{{{\rm{i}}}}}}}}Gx}{\sum }_{{{{{{{{{\bf{k}}}}}}}}}_{\parallel }n}{f}_{{{{{{{{{\bf{k}}}}}}}}}_{\parallel }n}|{\psi }_{{{{{{{{{\bf{k}}}}}}}}}_{\parallel }n}({{{{{{{\bf{R}}}}}}}}){|}^{2}$ at every step and adjusting the Fermi energy *E*_F_ to meet the condition that *n*_*G* = 0_ is equal to the unperturbed electron density *n*_0_.

### Optical response in the RPA

We start by writing the charge distribution *ρ*^ind^ induced in the semiconductor layer in the presence of an external electric potential *ϕ*^ext^ as *ρ*^ind^ = *χϕ*^ext^, where *χ* is the electric susceptibility and an overall e^−i*ωt*^ time dependence is understood. We now adopt the RPA^44^ to write $\chi={\chi }^{0}\cdot{(1-\upsilon\cdot {\chi }^{0})}^{-1}$ in terms of the noninteracting susceptibility ${\chi }^{0}({{{{{{{\bf{r}}}}}}}},{{{{{{{\bf{r}}}}}}}}^{\prime},\omega )=\delta (z)\delta (z^{\prime} ){\tilde{\chi }}^{0}({{{{{{{\bf{R}}}}}}}},{{{{{{{{\bf{R}}}}}}}}}^{\prime},\omega )$, where

8 $${\tilde{\chi }}^{0}({{{{{{{\bf{R}}}}}}}},\,{{{{{{{{\bf{R}}}}}}}}}^{\prime},\,\omega )=	 \frac{2{e}^{2}}{\hslash }\mathop{\sum}\limits_{{{{{{{{{\bf{k}}}}}}}}}_{\parallel }{{{{{{{{\bf{k}}}}}}}}}_{\parallel }^{\prime}n{n}^{\prime}}\frac{{f}_{{{{{{{{{\bf{k}}}}}}}}}_{\parallel }^{\prime}{n}^{\prime}}-{\,f}_{{{{{{{{{\bf{k}}}}}}}}}_{\parallel }n}}{\omega -{\varepsilon }_{{{{{{{{{\bf{k}}}}}}}}}_{\parallel }n}+{\varepsilon }_{{{{{{{{{\bf{k}}}}}}}}}_{\parallel }^{\prime}{n}^{\prime}}+{{{{{{{\rm{i}}}}}}}}{0}^{+}}\\ 	\times {\psi }_{{{{{{{{{\bf{k}}}}}}}}}_{\parallel }n}({{{{{{{\bf{R}}}}}}}}){\psi }_{{{{{{{{{\bf{k}}}}}}}}}_{\parallel }^{\prime}{n}^{\prime}}^{*}({{{{{{{\bf{R}}}}}}}}){\psi }_{{{{{{{{{\bf{k}}}}}}}}}_{\parallel }^{\prime}{n}^{\prime}}({{{{{{{{\bf{R}}}}}}}}}^{\prime}){\psi }_{{{{{{{{{\bf{k}}}}}}}}}_{\parallel }n}^{*}({{{{{{{{\bf{R}}}}}}}}}^{\prime}),$$

and obviously, the full susceptibility also bears an out-of-plane dependence as $$\chi ({{{{{{{\bf{r}}}}}}}},{{{{{{{\bf{r}}}}}}}}^{\prime},\omega )=\delta (z)\delta (z^{\prime} )\tilde{\chi }({{{{{{{\bf{R}}}}}}}},{{{{{{{{\bf{R}}}}}}}}}^{\prime},\omega )$$. Here, $$\upsilon ({{{{{{{\bf{r}}}}}}}},{{{{{{{\bf{r}}}}}}}}^{\prime} )$$ is the Coulomb potential produced at **r** by a unit charge placed at $${{{{{{{\bf{r}}}}}}}}^{\prime}$$, including the effect of screening by the surrounding environment. For simplicity, we assume the material in the ribbon array to be perfectly conducting at zero frequency, but invisible at the infrared optical frequencies under consideration (e.g., by embedding the entire structure in an index-matching medium), so that *υ* can be well approximated by the bare Coulomb potential $$\upsilon ({{{{{{{\bf{r}}}}}}}},{{{{{{{\bf{r}}}}}}}}^{\prime} )\,\approx \,1/|{{{{{{{\bf{r}}}}}}}}-{{{{{{{\bf{r}}}}}}}}^{\prime}|$$ in the calculation of *χ*. Now, given the ribbon array periodicity, we have $$\tilde{\chi }({{{{{{{\bf{R}}}}}}}},{{{{{{{\bf{R}}}}}}}}^{\prime},\omega )=\tilde{\chi }({{{{{{{\bf{R}}}}}}}}+\hat{{{{{{{{\bf{x}}}}}}}}}\ell a,{{{{{{{\bf{R}}}}}}}}^{\prime}+\hat{{{{{{{{\bf{x}}}}}}}}}\ell a,\omega )$$ for any integer $${\ell}$$, so we can write $$\tilde{\chi }({{{{{{{\bf{R}}}}}}}},{{{{{{{\bf{R}}}}}}}}^{\prime},\omega )={(2\pi )}^{-2}{\sum }_{G{G}^{\prime}}{{{{{{{{\rm{e}}}}}}}}}^{{{{{{{{\rm{i}}}}}}}}(Gx-{G}^{\prime}x^{\prime} )}\int\nolimits_{-\pi /a}^{\pi /a}d{q}_{x}\int\nolimits_{-\infty }^{\infty }d{q}_{y}\,{\tilde{\chi }}_{G{G}^{\prime}}({{{{{{{{\bf{q}}}}}}}}}_{\parallel },\omega )$$$${{{{{{{{\rm{e}}}}}}}}}^{{{{{{{{\rm{i}}}}}}}}{{{{{{{{\bf{q}}}}}}}}}_{\parallel }\cdot ({{{{{{{\bf{R}}}}}}}}-{{{{{{{{\bf{R}}}}}}}}}^{\prime})}$$, where the *q*_*x*_ integral is limited to the 1D 1BZ of the ribbon lattice and the components $${\tilde{\chi }}_{G{G}^{\prime}}({{{{{{{{\bf{q}}}}}}}}}_{\parallel },\omega )$$ are all we need to calculate the optical response to an external perturbation bearing an in-plane spatial dependence $$\propto {{{{{{{{\rm{e}}}}}}}}}^{{{{{{{{\rm{i}}}}}}}}{{{{{{{{\bf{q}}}}}}}}}_{\parallel }\cdot {{{{{{{\bf{R}}}}}}}}}$$. Finally, inserting the one-electron wave functions discussed above into Eq. (8), we readily find Eq. (3) in the main text.

### Thermal and electrical conductivities

An external static in-plane electric field **E** produces a 2D current density **j**^e^ = *σ*^DC^**E**, where *σ*^DC^ is the local DC (*ω* = 0) electrical conductivity tensor. Likewise, an in-plane temperature gradient induces a 2D thermal current density **j**^th^ = −*σ*^th^∇_**R**_*T*, where *σ*^th^ is the electronic thermal conductivity tensor. In the relaxation-time approximation, the effect of inelastic electron scattering enters via a phenomenological energy-independent damping time *τ*, contributed by different scattering channels such as impurities and acoustic phonons^53,54^, and we find the 2 × 2 tensors^49^

$${\sigma }^{{{{{{{{\rm{DC}}}}}}}}}=	 \frac{{e}^{2}\tau }{\hslash {\pi }^{2}}\mathop{\sum}\limits_{n}\int\nolimits_{-\pi /a}^{\pi /a}d{k}_{x}\int\nolimits_{0}^{\infty }d{k}_{y}\frac{{\nabla }_{{{{{{{{{\bf{k}}}}}}}}}_{\parallel }}\otimes {\nabla }_{{{{{{{{{\bf{k}}}}}}}}}_{\parallel }}{\varepsilon }_{{{{{{{{{\bf{k}}}}}}}}}_{\parallel }n}}{{{{{{{{{\rm{e}}}}}}}}}^{(\hslash {\varepsilon }_{{{{{{{{{\bf{k}}}}}}}}}_{\parallel }n}-{E}_{{{{{{{{\rm{F}}}}}}}}})/{k}_{{{{{{{{\rm{B}}}}}}}}}T}+1},\\ {\sigma }^{{{{{{{{\rm{th}}}}}}}}}=	 \frac{1}{{e}^{2}T}\left[{\sigma }^{(2)}-{\sigma }^{(1)}{\left({\sigma }^{{{{{{{{\rm{DC}}}}}}}}}\right)}^{-1}{\sigma }^{(1)}\right],$$

where we have defined

$${\sigma }^{{{{{(\alpha)}}}}}=	 \frac{{e}^{2}\tau }{{\pi }^{2}{k}_{{{{{{{{\rm{B}}}}}}}}}T}\mathop{\sum}\limits_{n}\int\nolimits_{-\pi /a}^{\pi /a}d{k}_{x}\int\nolimits_{0}^{\infty }d{k}_{y}{(\hslash {\varepsilon }_{{{{{{{{{\bf{k}}}}}}}}}_{\parallel }n}-{E}_{{{{{{{{\rm{F}}}}}}}}})}^{\alpha }\\ 	 \times ({{{{{{{{\bf{v}}}}}}}}}_{{{{{{{{{\bf{k}}}}}}}}}_{\parallel }n}\otimes {{{{{{{{\bf{v}}}}}}}}}_{{{{{{{{{\bf{k}}}}}}}}}_{\parallel }n})\frac{{{{{{{{{\rm{e}}}}}}}}}^{(\hslash {\varepsilon }_{{{{{{{{{\bf{k}}}}}}}}}_{\parallel }n}-{E}_{{{{{{{{\rm{F}}}}}}}}})/{k}_{{{{{{{{\rm{B}}}}}}}}}T}}{{\left[{{{{{{{{\rm{e}}}}}}}}}^{(\hslash {\varepsilon }_{{{{{{{{{\bf{k}}}}}}}}}_{\parallel }n}-{E}_{{{{{{{{\rm{F}}}}}}}}})/{k}_{{{{{{{{\rm{B}}}}}}}}}T}+1\right]}^{2}},$$

with ${{{{{{{{\bf{v}}}}}}}}}_{{{{{{{{{\bf{k}}}}}}}}}_{\parallel }n}={\nabla }_{{{{{{{{{\bf{k}}}}}}}}}_{\parallel }}{\varepsilon }_{{{{{{{{{\bf{k}}}}}}}}}_{\parallel }n}$ being the electron group velocity^49^. Equations (4) and (5) in the main text follow directly from these expressions by using the electron dispersion in Eq. (2b).

## Supplementary information

Supplementary Information

## Data Availability

The data that support the findings of this study are readily generated from the presented self-contained formalism and are also available from the corresponding author upon reasonable request.
