# Supplementary material for: An image interaction approach to quantum-phase engineering of two-dimensional materials
Source: Nat Commun. 2022 Sep 2;13:5175. doi: 10.1038/s41467-022-32508-5 (PMC9440131; doi:10.1038/s41467-022-32508-5)
Supplement: Supplementary file 1 — Supplementary Information [file 41467_2022_32508_MOESM1_ESM.pdf]

An image interaction approach to quantum-phase engineering of two-dimensional materials

– SUPPLEMENTARY INFORMATION –

Valerio Di Giulio,[1] P. A. D. Gonçalves,[1] and F. Javier García de Abajo[1,2,*]

[1]*ICFO-Institut de Ciencies Fotoniques, The Barcelona Institute of Science and Technology, 08860 Castelldefels (Barcelona), Spain*

[2]*ICREA-Institució Catalana de Recerca i Estudis Avançats, Passeig Lluís Companys 23, 08010 Barcelona, Spain*

Contents

Supplementary Note 1. Calculation of single-particle electron states 1

Supplementary Note 2. Quantification of the image energy 2

Supplementary Note 3. Probing the optical response through EELS 3

Supplementary Note 4. Q-phase graphene 6

Supplementary References 6

Supplementary Note 1. CALCULATION OF SINGLE-PARTICLE ELECTRON STATES

We consider a semiconductor monolayer under the conditions described in the main text, characterized by a parabolic conduction band of effective mass m^*, partially filled to a Fermi level E_F^0. Conduction electrons can then be labeled by the in-plane wave vector $\mathbf{k}_\parallel = (k_x, k_y)$, such that the electron energies are $\hbar\varepsilon_{\mathbf{k}_\parallel} = \hbar^2 k_\parallel^2/2m^*$ relative to the bottom of the conduction band. We also incorporate a periodic image potential produced by interaction with a neighboring conductive ribbon array of parameters and material composition as described in the main text (see Fig. 1b in the main text). The image potential landscape is introduced through a term $V^{\rm im}(x) = -V_0 p(x)$ in the electron Hamiltonian, where $V_0 > 0$ measures the magnitude of the image interaction and $p(x) = \sum_{\ell=-\infty}^{\infty} \theta(\ell a + b - x)\theta(x - \ell a)$ follows the profile of the ribbon array (width b, period a) by means of step functions. Electron bands are then emerging, so we restrict k_x to the 1BZ and introduce a band index n to label electron states by $(\mathbf{k}_\parallel, n)$ with $|k_x| < \pi/a$. We only consider in-plane electron motion (i.e., in the $\mathbf{R} = (x, y)$ plane), under the assumption that electron states are tightly confined along the out-of-plane direction, such that their wave functions can be factorized as $\psi_{\mathbf{k}_\parallel n}(\mathbf{R})\psi_\perp(z)$, where $\psi_\perp(z)$ is shared by all states. In addition, we approximate the out-of-plane probability density as $|\psi_\perp(z)|^2 \approx \delta(z)$ (i.e., the 2D material limit). The remaining in-plane components are governed by the Schrödinger equation

$$\mathcal{H}(\mathbf{R})\psi_{\mathbf{k}_\parallel n}(\mathbf{R}) = \hbar\varepsilon_{\mathbf{k}_\parallel n}\psi_{\mathbf{k}_\parallel n}(\mathbf{R}), \tag{1}$$

with the Hamiltonian

$$\mathcal{H}(\mathbf{R}) = \mathcal{H}^0(\mathbf{R}) + V^{\rm im}(x) + V^{\rm H}(\mathbf{R}), \tag{2}$$

where $\mathcal{H}^0(\mathbf{R}) = -\hbar^2\nabla_{\mathbf{R}}^2/2m^*$ and we introduce electron Coulomb repulsion through the Hartree potential[1] $V^{\rm H}(\mathbf{R}) = e^2 \int d^2\mathbf{R}' \left[n(\mathbf{R}') - n_0\right]/|\mathbf{R} - \mathbf{R}'|$, where $n(\mathbf{R}) = 2\sum_{\mathbf{k}_\parallel n} f_{\mathbf{k}_\parallel n}|\psi_{\mathbf{k}_\parallel n}(\mathbf{R})|^2$ is the electron density, the factor of 2 accounts for spin degeneracy, and $f_{\mathbf{k}_\parallel n}$ is the Fermi–Dirac distribution. Here, we calculate the electronic band structure at zero temperature, such that $f_{\mathbf{k}_\parallel n} = \theta(E_F - \hbar\varepsilon_{\mathbf{k}_\parallel n})$, where the Fermi energy E_F is adjusted to make the average electron density equal to that of the unperturbed 2D semiconductor n_0. Then, E_F depends on the applied image potential and generally differs from E_F^0. We solve Eq. (1) iteratively by calculating the Hartree potential at each step, fixing E_F to preserve the average electron density, and mixing the new Hartree potential with the previous one until convergence is achieved after a few iterations.

Because of the periodicity of the Hamiltonian (i.e., $\mathcal{H}(\mathbf{R}) = \mathcal{H}(\mathbf{R} + \ell a\hat{\mathbf{x}})$ for any integer ℓ), the eigenstates can be written as $\psi_{\mathbf{k}_\parallel n}(\mathbf{R}) = e^{i\mathbf{k}_\parallel\cdot\mathbf{R}} u_{\mathbf{k}_\parallel n}(\mathbf{R})/L$ (Bloch's theorem), where L^2 is the semiconductor area, and the functions

$u_{\mathbf{k}_\parallel n}(\mathbf{R})$ also satisfy $u_{\mathbf{k}_\parallel n}(\mathbf{R}+\ell\hat{\mathbf{x}}) = u_{\mathbf{k}_\parallel n}(\mathbf{R})$ for any integer ℓ. In addition, since the image potential only depends on x, we can factorize the wave functions and separate the energies as

$$\psi_{\mathbf{k}_\parallel n}(\mathbf{R}) = \mathrm{e}^{\mathrm{i}\mathbf{k}_\parallel\cdot\mathbf{R}} u_{k_x n}(x)/L, \tag{3a}$$

$$\hbar\varepsilon_{\mathbf{k}_\parallel n} = \hbar\varepsilon^x_{k_x n} + \hbar^2 k_y^2/2m^*, \tag{3b}$$

where we have plane waves in the direction of translational invariance y. Along the direction of periodicity x, we find $\varepsilon^x_{k_x n}$ and $u_{k_x n}(x)$ by solving the 1D problem

$$\left[\hbar^2(k_x - \mathrm{i}\partial_x)^2/2m^* + V^{\mathrm{im}}(x) + V^{\mathrm{H}}(x)\right] u_{k_x n}(x) = \hbar\varepsilon_{k_x n} u_{k_x n}(x), \tag{4}$$

where we introduced the 1D Hartree potential

$$V^{\mathrm{H}}(x) = -2e^2 \int_{-\infty}^{\infty} dx' \left[n(x') - n_0\right] \log|x - x'|, \tag{5}$$

and $n(x) = (1/\pi^2)\sum_n \int_{-\pi/a}^{\pi/a} dk_x \int_0^\infty dk_y\, |u_{k_x n}(x)|^2\, \theta(E_{\mathrm{F}} - \hbar\varepsilon^x_{k_x n} - \hbar^2 k_y^2/2m^*)$ is the electron density profile. To obtain Eq. (5), we have employed the prescription $\sum_{\mathbf{k}_\parallel} \to (L/2\pi)^2 \int d^2\mathbf{k}_\parallel$, used the integral and limit $\int_0^b dy/\sqrt{(x-x')^2+y^2} = \log[b + \sqrt{(x-x')^2+b^2}] - \log|x-x'| \xrightarrow[b\to\infty]{} \log(2b) - \log|x-x'|$, and applied the condition of charge neutrality to eliminate x-independent terms. Now, moving to Fourier space, we transform Eq. (4) into a linear system of equations,

$$\left[\frac{\hbar^2}{2m^*}(k_x+G)^2 - \hbar\varepsilon^x_{k_x n}\right] u_{k_x n,G} + \sum_{G'} \left(V^{\mathrm{im}}_{G-G'} + V^{\mathrm{H}}_{G-G'}\right) u_{k_x n,G'} = 0, \tag{6}$$

where G and G' are reciprocal lattice vectors (i.e., multiples of $2\pi/a$). This equation involves the Fourier coefficients of different quantities, defined through the relations $f_G = (1/a)\int_0^a dx\, f(x)\mathrm{e}^{-\mathrm{i}Gx}$ and $f(x) = \sum_G f_G \mathrm{e}^{\mathrm{i}Gx}$. In particular, from the stepwise profile of the image potential (see above), we find

$$V^{\mathrm{im}}_G = V_0 \times \begin{cases} (\mathrm{i}/aG)\left(1 - \mathrm{e}^{-\mathrm{i}Gb}\right), & \text{for } G \neq 0, \\ -b/a, & \text{for } G = 0. \end{cases}$$

Likewise, from Eq. (5), the coefficients of the Hartree potential reduce to

$$V^{\mathrm{H}}_G = 2\pi e^2 \times \begin{cases} n_G/|G|, & \text{for } G \neq 0, \\ 0, & \text{for } G = 0, \end{cases}$$

where we have used the integral $\int_{-\infty}^{\infty} dx\, \mathrm{e}^{\mathrm{i}Gx} \log|x| = -\pi/|G|$ (see Eq. (4.441-2) in Ref.[2]).

Supplementary Note 2. QUANTIFICATION OF THE IMAGE ENERGY

We intend to quantify the influence of the image interaction on the electronic behavior in the semiconductor considering the different parameters that define the system. We start from the density $n = k_{\mathrm{F}}^2/2\pi$ and the kinetic energy $E^{\mathrm{kin}} = L^2\pi\hbar^2 n^2/2m^*$ of a 2DEG. Following a density functional theory approach in the local-density approximation, we write the total energy as a functional of the electronic density $n(\mathbf{R})$:

$$E[n] = \frac{\pi\hbar^2}{2m^*}\int d^2\mathbf{R}\, n^2(\mathbf{R}) + \int d^2\mathbf{R}\, V^{\mathrm{im}}(\mathbf{R}) n(\mathbf{R}) + \frac{e^2}{2}\int d^2\mathbf{R} d^2\mathbf{R}' \,\frac{\Delta n(\mathbf{R})\Delta n(\mathbf{R}')}{|\mathbf{R}-\mathbf{R}'|}, \tag{7}$$

where $\Delta n(\mathbf{R}) = n(\mathbf{R}) - n_0$. The ground-state density in the many-electron system is then obtained by minimizing Eq. (7), subject the constraint $\int d^2\mathbf{R}\, \Delta n(\mathbf{R}) = 0$. By introducing a Lagrange multiplier λ and imposing the vanishing of the functional derivative with respect to n (i.e., $\delta_n E[n] = \lambda$), we find

$$\frac{\pi\hbar^2}{m^*}[\Delta n(\mathbf{R}) + n_0] + V^{\mathrm{im}}(x) + \frac{e^2}{2}\int d^2\mathbf{R}' \,\frac{\Delta n(\mathbf{R}')}{|\mathbf{R}-\mathbf{R}'|} = \lambda,$$

which, transforming all quantities to reciprocal space as in Sec. (**Supplementary Note 1**), can be written as

$$\frac{\pi\hbar^2}{m^*}\left[\Delta n(x)+n_0\right]+V^{\rm im}(x) \\ +e^2\pi\sum_{G\neq0}\frac{\Delta n_G}{|G|}{\rm e}^{{\rm i}Gx}=\lambda. \tag{8}$$

Now, as a crude approximation, we assume that $\Delta n(x)$ has the same periodicity and shape as $V^{\rm im}(x)$, oscillating between the values n_1 and $-n_1$ for $b=a/2$ so that the average electron density is conserved. By specifying Eq. (8) at two different points $0<x_1<b$ and $b<x_2<a$, and then subtracting the two resulting equations, we find the ratio

$$\frac{n_1}{n_0}=\frac{1}{2}\frac{V_0/{E_{\rm F}}^0}{1+\mathcal{A}[(V_{\rm C}/{E_{\rm F}}^0)/(k_{\rm BZ}/{k_{\rm F}}^0)^2]}, \tag{9}$$

where $\mathcal{A}=\sum_{G\neq0}(\pi^2/4a|G|^2)K_G({\rm e}^{{\rm i}Gx_1}-{\rm e}^{{\rm i}Gx_2})$, $K_G=\Delta n_G/n_1=(2{\rm i}/aG)({\rm e}^{-{\rm i}Gb}-1)$, and we use the coefficients $V_{\rm C}=e^2/a$ and $k_{\rm BZ}=\pi/a$ defined in the main text. We have verified that $\mathcal{A}$ evolves in the $(0,1)$ interval as the values of $x_1<x_2$ are varied. For instance, if $x_1=a/4$ and $x_2=3a/4$, we obtain $\mathcal{A}=\sum_{n=0}^{\infty}(-1)^n/(2n+1)^2\sim0.91$. We are interested in finding the ratio of the kinetic energy to the image potential energy, which in this approximation becomes

$$\left|\frac{E^{\rm kin}}{E^{\rm im}}\right|=\frac{\pi\hbar^2}{2m^*}\frac{\int dx\,n^2(x)}{\left|\int dx\,V^{\rm im}(x)n(x)\right|} \\ =\frac{{E_{\rm F}}^0}{2V_0}\frac{1+n_1^2/n_0^2}{|1+n_1/n_0|},$$

and finally, for small perturbations ($|n_1|\ll n_0$), it reduces to

$$\left|\frac{E^{\rm kin}}{E^{\rm im}}\right|\approx\left|\frac{{E_{\rm F}}^0}{2V_0}-\frac{1/4}{1+\mathcal{A}[(V_{\rm C}/{E_{\rm F}}^0)/(k_{\rm BZ}/{k_{\rm F}}^0)^2]}\right|. \tag{10}$$

The second fraction in the right-hand side of this equation can be neglected under the conditions investigated in the main text (i.e., for $V_{\rm C}/{E_{\rm F}}^0/(k_{\rm BZ}/{k_{\rm F}}^0)^2\gg1$), so the influence of the image potential on the material is simply quantified through the parameter $V_0/{E_{\rm F}}^0$.

Supplementary Note 3. PROBING THE OPTICAL RESPONSE THROUGH EELS

The optical response of the Q-phase materials under consideration can be probed through electron energy-loss spectroscopy (EELS). We follow the general methods discussed elsewhere[3] to calculate the loss probability for an electron moving with constant velocity $\mathbf{v}$ parallel to the semiconductor and oriented along in-plane directions either parallel or perpendicular to the ribbons. The electron is taken to be moving in vacuum on the side of the semiconductor that is not occupied by the ribbon structure used to produce the image potential (see insets in Supplementary Figure 1).

Adopting the electrostatic limit, the EELS probability $\Gamma_{\rm EELS}(\omega)$ is directly obtained from the screened Coulomb interaction $W(\mathbf{r},\mathbf{r}',\omega)$, which describes the potential produced at $\mathbf{r}$ by a unit point charge placed at $\mathbf{r}'$ and oscillating with frequency ω. More precisely[3],

$$\Gamma_{\rm EELS}(\omega)=\frac{e^2}{\pi\hbar}\int dt\int dt'\,{\rm e}^{{\rm i}\omega(t'-t)}\,{\rm Im}\left\{-W^{\rm ind}(\mathbf{r}_0+\mathbf{v}t,\mathbf{r}_0+\mathbf{v}t',\omega)\right\}, \tag{11}$$

where $\mathbf{r}_0$ describes the electron position at time $t=0$, and z_0 is the electron-surface separation. Now, from the analysis presented in the main text, defining $\mathbf{G}=G\hat{\mathbf{x}}$ with $G=2\pi m/a$ and m running over integer numbers, we consider points $z,z'>0$ above the semiconductor surface and express the screened interaction in terms of the Fourier components of the Fresnel reflection coefficient for p polarization:

$$W^{\rm ind}(\mathbf{r},\mathbf{r}',\omega)=-\frac{1}{2\pi}\int_{-\pi/a}^{\pi/a}dq_x\int_{-\infty}^{\infty}dq_y\sum_{GG'}\frac{r^{\rm p}_{GG'}(\mathbf{q}_\parallel,\omega)}{|\mathbf{q}_\parallel+\mathbf{G}'|}\,{\rm e}^{{\rm i}(\mathbf{q}_\parallel+\mathbf{G})\cdot\mathbf{R}}{\rm e}^{-{\rm i}(\mathbf{q}_\parallel+\mathbf{G}')\cdot\mathbf{R}'}{\rm e}^{-|\mathbf{q}_\parallel+\mathbf{G}|z}{\rm e}^{-|\mathbf{q}_\parallel+\mathbf{G}'|z'}. \tag{12}$$

Incidentally, we only write the induced part of the interaction because the direct Coulomb term does not contribute to Eq. (11).

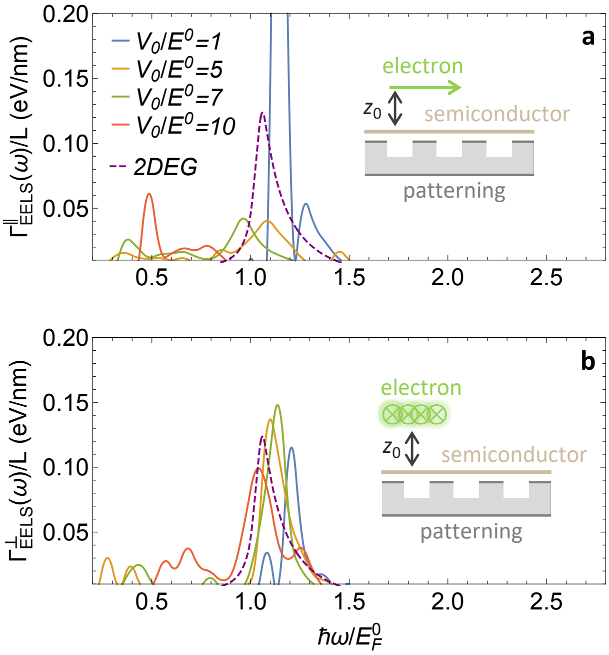

Supplementary Figure 1: **Probing the optical response of a Q-phase material through EELS.** (a) EELS probability experienced by a 50 eV electron moving parallel to the semiconductor under consideration at a distance $z_0 = 10\,$nm (see inset). We show results for different values of $V_0/E_{\rm F}^0$ (solid curves, obtained by inserting the loss function of Fig. 2 of the main text into Eq. (14)), compared with the probability calculated for a 2DEG described through the local Drude conductivity $\sigma(\omega) = (4\pi {\rm i} n_0/m^*)\big/(\omega + {\rm i}/\tau)$ (dashed curve). A phenomenological damping $\hbar/\tau = 10\,$meV is assumed in all cases. (b) Same as (a), but with the electron traveling parallel to the ribbons, and the probability averaged over lateral position x according to Eq. (13).

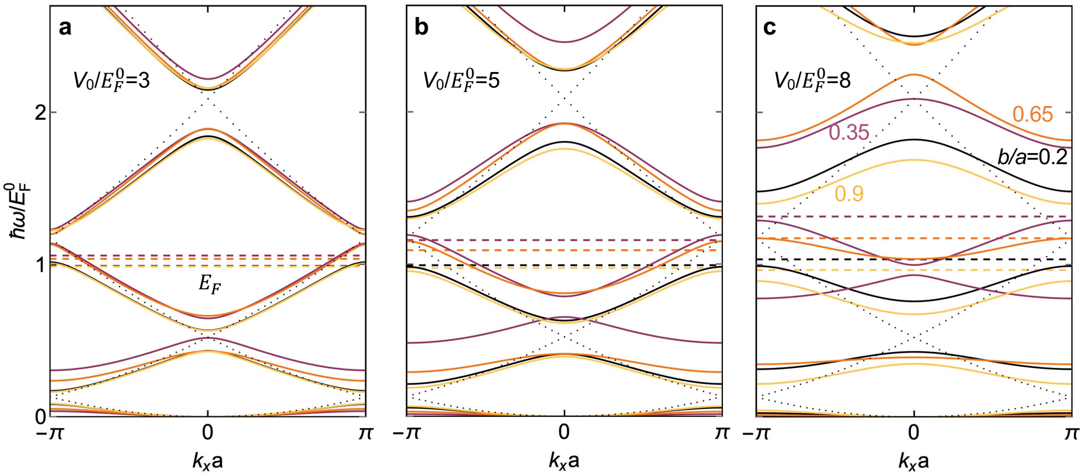

Supplementary Figure 2: **Modulation of the electronic band structure for different patterning ratios** b/a. We plot the electronic bands (solid curves) in the direction of periodic patterning for different values of b/a (see color-matched labels). Results are presented for $V_0/E_{\rm F}^0 = 3$ (a), $V_0/E_{\rm F}^0 = 5$ (b), and $V_0/E_{\rm F}^0 = 8$ (c), along with the corresponding normalized Fermi energies (dashed lines). Dotted curves stand for the 2DEG limit.

For an electron beam oriented along y (parallel to the ribbons), we take $\mathbf{r}_0 = (x_0, 0, z_0)$ and average over lateral beam positions x_0 across one period of the array, so that by inserting Eq. (12) into Eq. (11) and using the integral

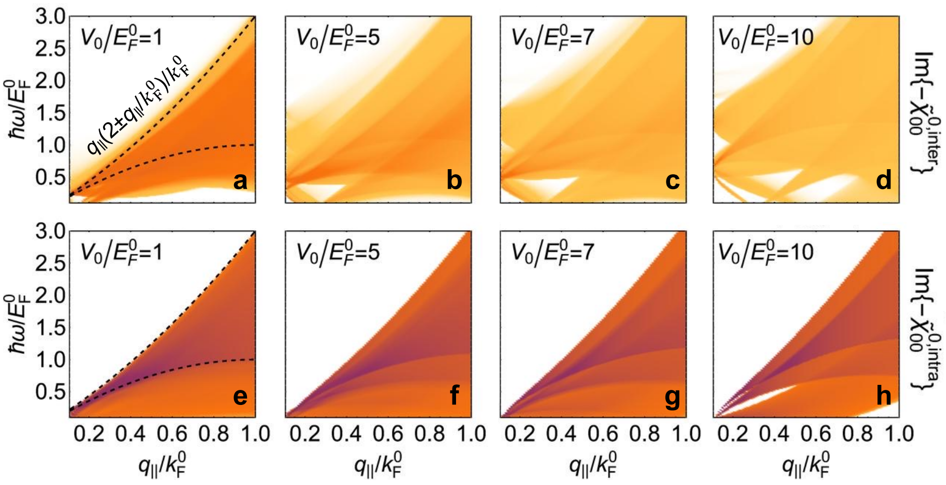

Supplementary Figure 3: **Interband and intraband contributions to single-particle excitations in Q-phase materials.** (a-d) Interband ($n \neq n'$ part of the sum in Eq. (3) of the main text) and (e-h) intraband ($n = n'$) contributions to the 2D noninteracting susceptibility $\tilde{\chi}^0_{00}$ as a function of transferred energy $\hbar\omega$ and in-plane wave vector $q_\parallel = |(q_x, q_y)|$, normalized to the Fermi energy $E^0_{\rm F}$ and the wave vector $k^0_{\rm F}$, respectively, under the same conditions as in Fig. 2 of the main text.

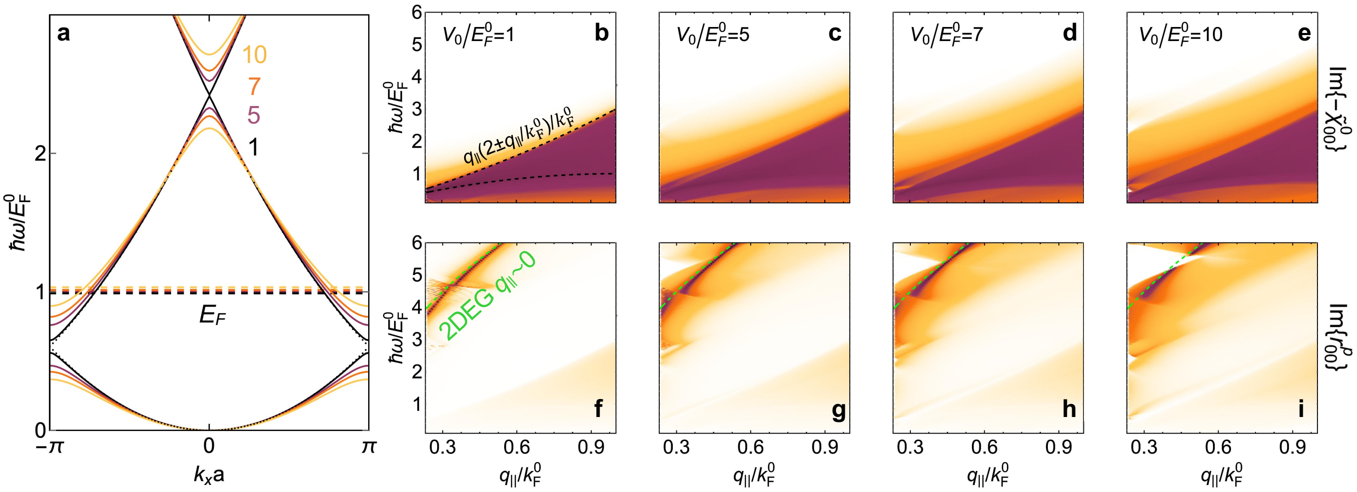

Supplementary Figure 4: **Effect of the effective mass on the electronic band structure and optical response in Q-phase materials.** We plot the same quantities as in Fig. 2 of the main text, but for a different set of parameters: $E^0_{\rm F} = 17\,$meV, $m^*/m_{\rm e} = 0.36$, $a = 10\,$nm, and $b = 5\,$nm.

$(1/a)\int_0^a dx_0\, {\rm e}^{{\rm i}(G-G')x_0} = \delta_{GG'}$, we find

$$\Gamma^{\perp}_{\rm EELS}(\omega) = \frac{2e^2L}{\pi\hbar v^2}\int_0^{\pi/a} dq_x \sum_G \frac{{\rm e}^{-2Q_1 z_0}}{Q_1}\,{\rm Im}\left\{r^{\rm p}_{GG}(q_x,\omega/v,\omega)\right\}, \tag{13}$$

where $Q_1 = \sqrt{(q_x+G)^2+\omega^2/v^2}$, L is the length of the electron trajectory, and the $\perp$ superscript refers to the fact that the beam is moving perpendicularly with respect to the direction of the array periodicity.

Likewise, for an electron traveling along the transverse ribbon direction x (i.e., parallel to the array periodicity), we can take $\mathbf{r}_0 = (0, 0, z_0)$ and insert Eq. (12) into Eq. (11) again to readily obtain

$$\Gamma^{\parallel}_{\rm EELS}(\omega) = \frac{2e^2L}{\pi\hbar v^2}\int_0^{\infty} dq_y \frac{{\rm e}^{-2Q_2 z_0}}{Q_2}\,{\rm Im}\left\{r^{\rm p}_{\tilde{G}\tilde{G}}(\tilde{q}_x,q_y,\omega)\right\}, \tag{14}$$

where $Q_2 = \sqrt{\omega^2/v^2+q_y^2}$, $\tilde{q}_x = \omega/v - \tilde{G}$, and $\tilde{G}$ is the only lattice vector satisfying the condition $|\tilde{q}_x| < \pi/a$. Obviously, $\tilde{G}$ may depend on ω/v, although we expect to have $\omega/v \ll \pi/a$, and therefore $G = 0$. In such scenario, Eq.

(14) coincides with the electrostatic limit of the probability obtained for an electron moving parallel to the surface of a photonic crystal[4].

In Supplementary Figure **1**, we present EELS spectra calculated by using Eqs. (13) and (14) for different strengths of the image interaction V_0 down to the 2DEG limit $V_0 = 0$ (dashed curves). Strong deviations are observed in the number and positions of the spectral features inherited from the dispersion relations presented in Fig. 2 in the main text. The in-plane anisotropy of the designed Q-phase material gives rise to substantially different profiles for the two electron beam orientations under consideration.

Supplementary Note 4. Q-PHASE GRAPHENE

Although thus far we have focused on a 2D semiconductor (i.e., a material hosting a conventional parabolic dispersion band), the introduced Q-phase material concept can be swiftly extended to other atomically thin material platforms including doped graphene (a 2D carbon allotrope in which charge carriers follow a linear energy-momentum dispersion[5]). In what follows, we explicitly illustrate such an extension. To that end, and since graphene charge carriers behave as massless Dirac fermions[5], the governing equation is (here for the sake of simplicity, we dismiss the Hartree electron-electron interaction)

$$\left[\mathcal{H}_0^{\rm g}(\mathbf{R}) + \mathcal{I}\,V^{\rm im}(x)\right]\Psi_{\mathbf{k}_\parallel ns}(\mathbf{R}) = \hbar\epsilon^{\rm g}_{\mathbf{k}_\parallel ns}\Psi^s_{\mathbf{k}_\parallel ns}(\mathbf{R}), \tag{15}$$

with the effective single-electron Hamiltonian $\mathcal{H}^{\rm g}(\mathbf{R}) = -{\rm i}\hbar v_{\rm F}\vec{\sigma}\cdot\nabla_{\mathbf{R}}$, which is valid near the $\mathbf{K}$ and $\mathbf{K}'$ points of the Brillouin zone of graphene. Here, $\vec{\sigma}$ are the Pauli matrices in the in-plane directions, $\mathcal{I}$ is the 2×2 identity matrix acting on the 2-component spinors $\Psi_{\mathbf{k}_\parallel ns}(\mathbf{R})$, and $v_{\rm F}\approx c/300$ is the Fermi velocity in graphene. Similarly to the case of the semiconductor, by expanding the solution of Eq. (15) $\Psi_{\mathbf{k}_\parallel ns}(\mathbf{R}) = \sum_{Gs} u_{\mathbf{k}_\parallel ns,G}\Psi^0_{\mathbf{k}_\parallel+G\hat{\mathbf{x}}s}(\mathbf{R})$ in the eigenstates of $\mathcal{H}^{\rm g}(\mathbf{R})$

$$\Psi^0_{\mathbf{k}_\parallel s}(\mathbf{R}) = \frac{{\rm e}^{{\rm i}\mathbf{k}_\parallel\cdot\mathbf{R}}}{\sqrt{2A}}\begin{pmatrix}1\\ s{\rm e}^{{\rm i}\theta_{\mathbf{k}_\parallel}}\end{pmatrix},$$

where $\theta_{\mathbf{k}_\parallel} = \arctan(k_x/k_y)$, we find the set of coupled equations

$$\left(s\hbar v_{\rm F}|\mathbf{k}_\parallel + G\hat{\mathbf{x}}| - \epsilon^{\rm g}_{\mathbf{k}_\parallel ns}\right)u_{\mathbf{k}_\parallel sn,G} + \sum_{G's'}\frac{V^{\rm im}_{G-G'}}{2}\left[1 + ss'{\rm e}^{{\rm i}\left(\theta_{\mathbf{k}_\parallel+G'\hat{\mathbf{x}}} - \theta_{\mathbf{k}_\parallel+G\hat{\mathbf{x}}}\right)}\right]u_{\mathbf{k}_\parallel s'n,G'} = 0. \tag{16}$$

This approach has been followed in several works[6–13] showing the inability of a scalar periodic potential to open band gaps, although it can lead to anisotropic group velocity renormalization as well as the creation of satellite Dirac cones. Once the band structure $\epsilon^{\rm g}_{\mathbf{k}_\parallel ns}$ is obtained from Eq. (16), the optical and transport properties can be computed in a similar way to that presented in the main text. In particular, the dependence of the electrical DC conductivity has been experimentally investigated by means of regional doping[14] as well as via either dielectric[15,16] or direct[17] tailoring of a gating potential, the latter showing an oscillatory behavior as a function of doping level.

Supplementary References

* Electronic address: javier.garciadeabajo@nanophotonics.es
1 L. Hedin and S. Lundqvist, in *Solid State Physics*, edited by D. T. Frederick Seitz and H. Ehrenreich (Academic Press, 1970), vol. 23 of *Solid State Physics*, pp. 1–181.
2 I. S. Gradshteyn and I. M. Ryzhik, *Table of Integrals, Series, and Products* (Academic Press, London, 2007).
3 F. J. García de Abajo, Rev. Mod. Phys. **82**, 209 (2010).
4 F. J. García de Abajo and L. A. Blanco, Phys. Rev. B **67**, 125108 (2003).
5 A. H. Castro Neto, F. Guinea, N. M. R. Peres, K. S. Novoselov, and A. K. Geim, Rev. Mod. Phys. **81**, 109 (2009).
6 C.-H. Park, L. Yang, Y.-W. Son, M. L. Cohen, and S. G. Louie, Nature Phys. **4**, 213 (2008).
7 C.-H. Park, L. Yang, Y.-W. Son, M. L. Cohen, and S. G. Louie, Phys. Rev. Lett. **101**, 126804 (2008).
8 M. Barbier, P. Vasilopoulos, and F. M. Peeters, Phys. Rev. B **80**, 205415 (2009).
9 L. Brey and H. A. Fertig, Phys. Rev. Lett. **103**, 046809 (2009).
10 L.-G. Wang and S.-Y. Zhu, Phys. Rev. B **81**, 205444 (2010).
11 M. Barbier, P. Vasilopoulos, and F. M. Peeters, Phys. Rev. B **81**, 075438 (2010).
12 S. Ono, M. Zhang, Y. Noda, and K. Ohno, J. Electron. Mater. **43**, 1505 (2013).

[13] S. Ono, J. Appl. Phys. **121**, 204301 (2017).
[14] K.-K. Bai, Y. Zhou, H. Z. L. Meng, H. Peng, Z.Liu, J.-C.Nie, and L. He, Phys. Rev. Lett. **113**, 086102 (2014).
[15] C. Forsythe, X. Zhou, K. Watanabe, T. Taniguchi, A. Pasupathy, P. Moon, M. Koshino, P. Kim, and C. R. Dean, Nature Nanotech. **13**, 566 (2018).
[16] Y. Li, S. Dietrich, C. Forsythe, T. Taniguchi, K. Watanabe, P. Moon, and C. R. Dean, Nature Nanotech. **16**, 525 (2021).
[17] M. Drienovsky, F.-X. S. A. Sandner, D. Weiss, J. Eroms, M.-H. Liu, F. Tkatschenko, and K. Richter, Phys. Rev. B **89**, 115421 (2014).
